# Relationship between Eating Behavior, Quality of Life and Weight Regain in Women after Bariatric Surgery

**DOI:** 10.3390/ijerph19137648

**Published:** 2022-06-22

**Authors:** Talita Nogueira Berino, Aline Leão Reis, Manuela Maria de Lima Carvalhal, Jeane Lorena Dias Kikuchi, Rachel Coêlho Ripardo Teixeira, Daniela Lopes Gomes

**Affiliations:** 1 Nucleus of Behavior Theory Research, Federal University of Pará, Belém 66075110, Pará, Brazil; a.leaoreis@gmail.com (A.L.R.); nutri.jeanedias@yahoo.com (J.L.D.K.); cripardo@ufpa.br (R.C.R.T.); danielagomes@ufpa.br (D.L.G.); 2 Nucleus of Tropical Medicine, Federal University of Pará, Belém 66075110, Pará, Brazil; manuela.carvalhall@gmail.com; 3 Nutrition Faculty, Federal University of Pará, Belém 66075110, Pará, Brazil

**Keywords:** quality of life, eating behavior, bariatric surgery, emotional eating

## Abstract

Individuals undergoing bariatric Surgery (BS) may have long-term weight regain. There is a need to investigate factors that may be related to this and if they can interfere with Quality of Life (QOL). This study aims to evaluate the relationship between eating behavior, perception of QOL, and weight regain in women after 24 months of bariatric surgery. This was a transversal study with 50 adult women residents in the city of Belém, Brazil. Sociodemographic, anthropometric, eating behavior (Three-Factor Eating Questionnaire—TFEQ-21) and perception of QOL (Item Short Form Healthy Survey—SF-36) data were collected. In 60% (*n* = 30) there was weight regain (≥15%), with a mean weight regain of 23.3% (±18.4). Emotional eating was the most frequent pattern (*p* = 0.047). Regarding QOL, the functional capacity and limitation due to physical aspects domains had a better perception (*p* < 0.0001). Women without weight regain showed a better perception of the functional capacity aspects (*p* = 0.007), limitation due to physical aspects (*p* = 0.044), social aspects (*p* = 0.048), and general physical components (*p* = 0.016) and also had an inverse association with the perception of QOL in physical components (*p* = 0.008). Patterns of eating behavior and weight regain can damage the perception of QOL, especially physical capacity. Long-term follow-up is essential to evaluate the behavior of people who have undergone BS in order to prevent weight regain and QOL damage.

## 1. Introduction

Bariatric surgery (BS) is the most suitable treatment for severe obesity. However, it promotes severe changes in the individual’s body, and may extend to psychological difficulties related to physiological and behavioral alterations [1]. It is common to see the effects of weight loss in the first months after surgery, although it is necessary to observe whether there is weight regain in long term, especially after 24 months postoperative [2]. There is no consensus on the proportion of weight regain considered harmful. Some studies use the proportion of 15% in relation to the lowest stable weight achieved after surgery as weight regain that can bring metabolic damage to the patient [3,4].

Generally, weight loss stabilizes within 1-year of the postoperative period. However, many factors can interfere with the maintenance of body weight and facilitate weight regain, especially after 24 months. Factors such as the level of hormones and the physiological adaptations that occur after surgery, in addition to factors related to non-adherence to the new lifestyle, changes in energy expenditure, level of physical activity, diet quality, eating behavior (such as grazing), and lack of psychological support [1,3,5].

BS also can interfere with Quality of Life (QOL). To evaluate QOL, studies have used the Medical Outcome Study 36—Item Short Form Healthy Survey (SF-36) [6], which, though not specific for BS, is more complete because it encompasses eight domains related to QOL [7,8]. Akkayaoğlu and Çelik [9], when evaluating QOL in patients who had undergone BS, observed an increase in the mean scores of all dimensions of the SF-36 in the postoperative period when compared to the period prior to surgery.

In the study by Jesus et al. [10], a tendency was observed, after 5-years of surgery, of higher scores for emotional eating among patients, being higher among those with weight regain. Another study, conducted only with women with more than 24 months of Roux-en-Y Gastric Bypass (RYGB), found that the higher the emotional eating scores, the lower the excess weight loss (EWL), regardless of surgery time [11].

Engström et al. [12] investigated changes in the perception of food control by comparing the preoperative period with reassessments after 1-year and 2-years of surgery, analyzing possible influences on weight loss and QOL. The group with good eating control showed better results in QOL in terms of social and mental health aspects and, after 2-years, showed significant improvement in general aspects of QOL, except in the domain of pain. The group with inadequate eating control showed more dysfunctional eating behaviors both 1-year and 2-years after surgery. However, in this study, there was no separation by gender, the participants were obese (body mass index—BMI > 50 kg/m^2^), they were evaluated only up to 2-years postoperative, and the surgical techniques included RYGB and Biliopancreatic Derivation.

In Brazil, obesity has increased every year, following the world trend, and in women the prevalence is higher than in men [13]. As with obesity, the number of bariatric surgeries performed has grown over the years, increased by 84.7% between 2011 and 2018, and women undergo more BS than men [14]. Regarding the records related to the type of surgery, in Brazil most surgeries are of the RYGB followed by sleeve [15].

Due to the facts presented, and due the scarcity of studies using these tools and in a postoperative period longer than 24 months in women, this study aims to test the association between the perception of QOL and the different eating patterns of women, also analyzing whether this relationship is influenced by the occurrence or not of weight regain and the time of surgery.

## 2. Materials and Methods

### 2.1. Type of Study and Participants

This was a cross-sectional study carried out on 50 women aged between 18 and 59 years old, with a minimum postoperative period of 24 months after BS (RYGB or Sleeve) and who agreed to participate in the research by signing the Informed Consent Form. Exclusion criteria: having undergone another type of surgery, having become pregnant after surgery, using illegal drugs, and living outside the metropolitan region of the city of Belém, in the state of Pará, in Brazil.

### 2.2. Data Collection

Participants were patients at a university hospital in a public university in the city of Belém, Brazil. Individual interviews were scheduled for data collection, held between June 2018 and February 2020. The interviews lasted approximately 1 h each and anthropometry was performed, in addition to the application of questionnaires. The responses were recorded in a specific survey form to be tabulated later.

### 2.3. Anthropometry

To measure current weight and height, a platform scale with a capacity of 300 kg and a graduation of 0.1 kg was used, with a 200 cm coupled stadiometer (precision of 0.5 cm) (Welmy^®^, Santa Bárbara d’Oeste, Brazil). Preoperative weight was reported by the participants, as was weight loss in the postoperative period. Preoperative BMI (kg/m^2^), current BMI (kg/m^2^), % excess weight loss (% EWL), and weight regain (kg and %) were calculated. BMI was calculated using the formula: weight (kg)/height^2^ (m^2^). To calculate the EWL (%), the equation was used: % EWL = weight loss in the postoperative period (kg) × 100/preoperative initial weight (kg)—ideal weight (kg), considering surgical success as an overweight loss (%) ≥ 50%. The ideal weight used in this formula was calculated using the method recommended by the Brazilian BS Consensus [16] according to the formula: Ideal weight (kg) = 53.975 + [(height in meters − 1.524) × 53.5433]. Weight regain (kg) was calculated from the subtraction of: current weight (kg)—lowest stable weight (kg) achieved in the postoperative period, which was later converted into a percentage (%) for analysis of the proportion of weight regain in relation to the lowest stable weight after surgery, ≥15% being considered significant weight regain.

### 2.4. Quality of Life

To measure QOL, the Item Short Form Healthy Survey (SF-36) was used, translated and validated into Portuguese [6]. The SF-36 includes 36 items on various aspects of perception and QOL related to health. The domains of this instrument are grouped into: Physical Component (PC) (domains of functional capacity, limitation due to physical aspects, pain and vitality); and Emotional Component (EC) (mental health domains, general health status, limitation due to emotional and social aspects). Each category varies from 2 to 10 items and all of them can be summarized into two components: the General Score of Physical Components and the General Score of Emotional Components. The results are expressed in a score on a scale from 0 to 100, in which 0 corresponds to the worst perception and 100 to the best perception of QOL.

### 2.5. Eating Behavior

Participants completed the 21-item self-report Three-Factor Eating Questionnaire (TFEQ-21) [17], which assesses attitudes related to eating behavior in obese adult women and in BS through three dimensions of eating behavior: emotional eating, cognitive restriction, and uncontrolled eating. The TFEQ-21 can be used in women with obesity and for BS patients [17]. Self-reports were identified based on responses on a Likert scale with four options: 1—totally false; 2—false most of the time; 3—true most of the time; 4—totally true. Scores can vary from 0 to 100, the higher the score, the more the behavioral pattern is present in the individual’s repertoire.

### 2.6. Ethical Aspects

This study was approved by the Human Research Ethics Committee (opinion No. 2.170.863), complying with the legal requirements of Resolutions 466/12 and 510/16 of the Brazilian National Health Council and the Declaration of Helsinki.

### 2.7. Data Analysis

The Statistical Package for Social Sciences software, version 24.0, was used for all the analyses. Descriptive results were expressed as measures of central tendency and dispersion. The Kolmogorov–Smirnov test was carried out to assess normality. For comparison between groups, the Mann–Whitney test was used, and for intra-group comparisons, the Kruskal–Wallis test was carried out. In these tests, the sample was divided into two categories based on the presence and absence of weight regain. The covariate correlations were performed using the Spearman correlation test, and those variables that showed statistical significance in the covariate analysis were inserted in a multiple linear regression model (statistical significance *p* < 0.05).

## 3. Results

Fifty women participated, aged between 21 and 59 years old, with an average of 40 (±11.4) years old, the majority single (56.0%), with an average education level of 14 years (±2.5). The monthly average income was BRL 3052.80 (±1755.90) (USD 645.55 ± 371.30, dollar quote in 2022).

About 68% of the women underwent RYGB and 32% sleeve, and 60% had significant weight regain, with an average of 23.3% (±18.4). The postoperative period was, on average, 62 months (61.9 ± 47.2), the mean BMI in the preoperative period was 44.0 kg/m^2^ (±6.6), and the current average BMI was 29.7 kg/m^2^ (±5.4). The average excess weight loss was 75.6% (±28.8).

Emotional eating (*p* = 0.047) was the most frequent eating behavior observed. Regarding QOL, the domains with the best perception obtained were the functional capacity and limitation to physical aspects, demonstrating a perception of good quality in aspects related to movement and physical activity. The worst perceptions were in the domains of pain and vitality, demonstrating pain during daily activities and low vigor and motivation (*p* < 0.0001) (Table 1).

Table 2 shows the eating behavior and QOL according to the presence or absence of weight regain in the women in the study. In the group without weight regain, the emotional eating score was significantly higher (*p* = 0.048) than cognitive restriction and uncontrolled eating. Nevertheless, there was no significant difference between groups when comparing the domains of eating behavior (Table 2).

In both groups, the functional capacity domain was the one that stood out the most, with a significantly higher score (*p* = 0.0001). In the comparison between the groups, statistically significant differences were found in the scores on functional capacity (*p* = 0.007), limitation by physical aspects (*p* = 0.044) and social aspects (*p* = 0.048), and general physical QOL component (*p* = 0.016), suggesting that weight regain may be a factor that contributes to a worse perception of QOL (Table 2).

In the correlational analysis (Table 3), the data demonstrate significant associations between quality of life, eating behavior, weight regain, excess weight loss and time after surgery. It was found the following significant direct associations with the time of surgery: current weight (ρ^2^ = 0.310; *p* = 0.014) and weight regain (ρ^2^ = 0.528; *p* <0.0001). The variables inversely correlated with weight regain, observed functional capacity (ρ^2^ = −0.371; *p* = 0.004), limitation due to physical aspects (ρ^2^ = −0.424; *p* = 0.001), pain (ρ^2^ = −0.254; *p* = 0.038) and social aspects (ρ^2^ = −0.255; *p* = 0.037), This suggests that the greater the weight regain, the worse the perception of QOL in these aspects.

The scores in the domain of uncontrolled eating had a direct correlation with weight regain (ρ^2^ = 0.272; *p* = 0.028) and current weight (ρ^2^ = 0.263; *p* = 0.032); in addition, an inverse correlation was observed with the scores of functional capacity (ρ^2^ = −0.356; *p* = 0.006), social aspects (ρ^2^ = −0.259; *p* = 0.035), general physical component (ρ^2^ = −0.272; *p* = 0.028), and general emotional component (ρ^2^ = −0.270; *p* = 0.029) of QOL (Table 3).

There was correlation between weight regain and the domain of uncontrolled eating (*p* = 0.045), which remained significant in linear regression. In addition, to show the statistical significance of uncontrolled eating (*p* = 0.041) when included the time of surgery in a second model, suggesting that this correlation remains independent of the time of surgery (Table 4).

A second linear regression points out that the weight regain was directly correlated with the physical QOL component (*p* = 0.001). When the variable time of surgery was included in the second model, the correlation found in model 1 maintained statistical significance (*p* = 0.008), however, the time of surgery was not significant in QOL (*p* = 0.528) (Table 5).

It was evaluated whether there was a difference in the profile of eating pattern and QOL score of the participants according to the surgical technique performed, however, no statistically significant difference was found.

## 4. Discussion

The results pointed out that eating behavior, QOL, and weight regain were interconnected in the women studied. Weight stabilization is expected to occur between 12 and 18 months after surgery, and it is natural that there is a recovery of part of the lost weight [18,19]. However, more than half of the sample presented weight regain, with the average proportion of this regain greater than 20%, which is considered significant in Brazilian studies [5,20].

Emotional eating was the most present eating behavior and it was also the highest score in the group without regain. When comparing the score of eating patterns between groups based on weight regain, there was no difference between them, as showed in the study by Silva et al. [5], performed with 80 patients after 24 months of RYGB.

There was a significant correlation between weight regain and uncontrolled eating. Some studies did not find these results [21,22], though they were carried out up to 2-years after BS. Engström et al. [12], on the other hand, found that the group with uncontrolled eating had a reduction in emotional eating 1 year after BS, but after 2-years it returned to the same level as the preoperative period. The group that did not present uncontrolled eating had a reduction, over two years, of emotional eating and increased cognitive restriction. There is still no single pattern of eating behavior in patients who underwent BS, which suggests the need for further studies, especially with a qualitative methodology.

Functional capacity and limitation due to physical aspects were the domains of QOL with the highest scores, indicating a better function in these aspects by the participants. Other studies suggested that BS is able to improve the perception of QOL in the individual, since the significant reduction in weight allows achievements both in the physical and emotional spheres that were previously made impossible by overweight [12,23,24].

Women without weight regain had higher scores on physical components in general, functional capacity, limitation due to physical aspects, and social aspects. This result is in line with that of Perdue et al. [25], in that the differences in SF-36 were significant in the domains of vitality, mental health, and summary of emotional components, all of which were smaller in women who still considered themselves obese, which may mean that BS and weight loss as a result of it are processes that are too fast for the brain and they have an identification of themselves as obese even if the body no longer corresponds to this.

It was observed that pain was the domain with the lowest score, demonstrating impairment of activities due to pain. The study by Høgestøl et al. [26] found that a considerable part of the patients who underwent RYGB after 5-years of the surgery still had abdominal pain and this interfered with the perception of QOL. In the study by Laurino Neto, and Herbella [27], using the SF-36, it was found that in the short-term postoperative period there was an improvement in pain, however, after 7-years the score for this domain decreased again. The authors list the possible explanations: weight regain, aging and presence or recurrence of comorbidities.

The time of surgery was correlated to weight regain increases, decreasing the excess weight loss and the perception of QOL. After all, the more weight the person regains, the more difficulties are reported. The relationship between time of surgery and weight loss was also found in a survey that evaluated 50 adults (72.5% women) in 1-year and 5-years after sleeve and found a significant weight regain average in the fifth postoperative year when compared to the first year, in which the average % EWL decreased [28].

There are many factors that work simultaneously influencing weight loss and maintaining the success of BS [29]. In the first 2-years after BS, the patient is more likely to follow nutritional recommendations, the reduction in the amount of food eaten is greatly influenced by anatomy and physiological changes, there is an increase in the feeling of well-being and a decrease in possible psychopathologies. Nonetheless, after this period, new changes in behavior occur, with a tendency to recover lost weight [19,20]. That is, the time of surgery and the poor quality of food can be predictive factors for regain after 24 months of surgery [5,30], though they can also influence weight regain a practice of physical activities and anatomy and physiological changes [5], besides psychological, metabolic, hormonal issues and even complications arising from the surgical procedure [31]. The study by Rocha, Hociko and Oliveira [20] found that the factors most associated with regaining weight were inadequate nutrition, lack of physical activity and lack of nutritional monitoring in the postoperative period.

The increase in lack of control eating seems to increase weight regain and to decrease the perception of QOL components. Devlin et al. [7] found that the lack of eating control was associated with lower weight loss and long-term weight regain (7-years) in people undergoing RYGB, which could compromise the results of the surgery. Wiedemann, Ivezaj, and Grilo [32] pointed out that the presence of uncontrolled eating and emotional eating may be predictors of worse results related to postoperative weight, though, the instrument used by them was not the TFEQ-21 and they analyzed only patients submitted to the sleeve less than 1-year after the surgery.

There was also an inverse relationship between weight regain and the perception of physical components of QOL. In a systematic review and meta-analysis including 82 studies in which QOL was analyzed (the most used was SF-36) before and after BS, inverse and significant relationships were found between BMI and QOL [33]. This result reinforces that even with improvements, the mental/social components are always below the physical components. Probably the improvement in QOL occurs due to weight reduction and remission of associated diseases.

This study is significant when evaluating the participants in the long-term postoperative period, since that in this later period there is a greater chance of weight regain due to several factors, and consequently, a decrease in quality of life. The correlation between lack of uncontrolled eating and weight regain found suggests the importance of this study in evaluating the eating behavior of patients in the late postoperative period, whereas many articles assess these aspects only in the immediate postoperative period. In addition, it is noteworthy that the results related to the perception of quality of life, both in physical and emotional aspects, demonstrate how fundamental it is to maintain long-term psychological follow-up.

This study has limitations such as the small sample size, the lack of a specific questionnaire for the targeted public and also the failure to monitor the women studied. Therefore, it is not possible to generalize these results. New studies with a larger number of participants, that further explore the variables studied, in addition to including new variables, are needed. In addition, it is also necessary to develop and validate specific questionnaires for people undergoing BS, including eating behavior and QOL, to assess the long-term follow-up of these people, both for research purposes but also in clinical practice. In addition, further studies are suggested to assess eating behavior and perception of quality of life in less invasive surgical techniques such as endovascular bariatric surgery [34]. Studies that investigate behavioral patterns that can predispose to eating disorders are also suggested to define the best surgical technique, as well as to carry out multiprofessional monitoring after surgery, in the short and long term.

Despite the limitations, the study is relevant to contribute scientifically to the understanding of the processes that permeate weight regain and eating patterns in women submitted to long-term BS and the influence on their QOL.

## 5. Conclusions

The most frequent pattern of eating behavior was emotional eating in all women. The perception of QOL was relatively higher in relation to physical aspects, demonstrating that there is a better perception of functional capacity and that there are fewer limitations caused by physical assignments. Women who did not present weight regain expressed a better perception of QOL. There was an association between weight regain and decreased perception of the physical components of QOL, just as weight regain was associated with the presence of uncontrolled eating, a relationship strengthened by the increase in the time of surgery. It is concluded that relevant weight regain occurred after 24 months of BS in the women studied. This weight regain had an influence on eating behavior and consequently in the perception of QOL. Nutritional and psychological monitoring is essential in the long term for people who underwent BS.

## Figures and Tables

**Table 1:** Eating behavior and perception of quality of life in women with more than 24 months after bariatric surgery.

	Mean ± SD	Range	*p*-Value *
		Minimum–Maximum	
Eating behavior			
Cognitive restriction	48.7 ± 19.8	0.0–83.0	0.047
Emotional eating	57.5 ± 28.9	0.0–100.0	
Uncontrolled eating	46.9 ± 26.5	4.0–100.0	
QOL			
Functional capacity	78.8 ± 18.1	25.0–100.0	<0.0001
Limitation due to physical aspects	72.5 ± 37.2	0.0–100.0	
Pain	56.6 ± 24.5	0.0–100.0	
General health status	60.3 ± 15.8	25.0–87.0	
Vitality	57.3 ± 20.7	0.0–90.0	
Social aspects	68.5 ± 25.9	12.5–100.0	
Limitation due to emotional aspects	67.3 ± 42.9	0.0–100.0	
Mental Health	68.0 ± 17.0	28.0–100.0	

* Kruskal–Wallis/SD test = standard deviation.

**Table 2:** Eating behavior and QOL according to the presence or absence of weight regain in women with more than 24 months of BS.

	Weight Regain (*n* = 50)				*p*-Value *
	Absent (*n* = 20)		Present (*n* = 30)		
Eating Behavior	Mean ± SD	Median (P5–P95)	Mean ± SD	Median (P5–P95)	
Cognitive restriction	53.4 ± 19.9	58.5 (44.1–62.7)	45.6 ± 19.5	47.2 (38.3–52.9)	0.093
Emotional eating	58.4 ± 27.5	67.0 (45.5–71.2)	56.9 ± 30.3	53.0 (45.6–68.2)	0.984
Uncontrolled eating	39.2 ± 26.0	26.5 (27.0–51.3)	52.0 ± 26.0	50.0 (42.3–61.7)	0.069
*p*-value **	0.048		0.393		
**QOL**	**Mean ± SD**	**Median (P5–P95)**	**Mean ± SD**	**Median (P5–P95)**	*p*-value *
General score of physical components	74.0 ± 11.7	76.1 (68.5–79.5)	62.4 ± 18.1	68.9 (55.7–69.2)	0.016
Functional capacity	86.5 ± 14.8	87.5 (79.6–93.4)	73.7 ± 18.5	75.0 (66.8–80.6)	0.007
Limitation due to physical aspects	83.8 ± 32.7	100.0 (68.4–99.1)	65.0 ± 38.6	75.0 (50.6–79.4)	0.044
Pain	61.7 ± 21.5	62.0 (51.7–71.7)	53.2 ± 26.1	51.0 (43.5–63.0)	0.251
General health status	64.1 ± 13.4	67.0 (57.8–70.3)	57.8 ± 17.0	64.5 (51.4–64.2)	0.205
General score of emotional components	68.6 ± 18.6	76.0 (59.9–77.3)	63.1 ± 20.8	66.9 (55.3–70.9)	0.332
Vitality	61.8 ± 15.8	61.8 (54.3–69.2)	54.3 ± 23.1	57.5 (45.7–63.0)	0.296
Social aspects	76.3 ± 26.3	87.5 (64.0–88.5)	63.3 ± 24.8	62.5 (54.1–72.6)	0.048
Limitation due to emotional aspects	71.1 ± 39.4	100.0 (53.2–90.1)	64.4 ± 45.4	100.0 (47.5–81.4)	0.608
Mental health	64.6 ± 16.5	64.0 (56.9–72.3)	70.3 ± 17.3	72.0 (63.8–76.7)	0.218
*p*-value **	0.0001		0.0001		

* Mann–Whitney test, ** Kruskal–Wallis test.

**Table 3:** Correlation between time after surgery, quality of life, eating behavior, and anthropometry of women with more than 24 months of bariatric surgery.

	ρ^2^	*p*-Value *
Time after surgery (months)		
Current weight	0.310	0.014
Excess Weight Loss (%)	−0.238	0.048
Weight regain (kg)	0.528	<0.0001
Functional capacity	−0.424	0.001
Limitation due to physical aspects	−0.274	0.027
Excess Weight Loss (%)		
Functional capacity	0.272	0.028
Limitation due to physical aspects	0.345	0.007
Vitality	0.246	0.043
Limitation due to emotional aspects	0.271	0.028
Weight regain (kg)		
Functional capacity	−0.371	0.004
Limitation due to physical aspects	−0.424	0.001
Pain	−0.254	0.038
Social Aspects	−0.255	0.037
Uncontrolled eating		
Current weight	0.263	0.032
Weight regain (kg)	0.272	0.028
Functional capacity	−0.356	0.006
Social Aspects	−0.259	0.035
Physical Components of QOL	−0.272	0.028
Emotional Components of QOL	−0.270	0.029

* Spearman correlation test.

**Table 4:** Correlation between weight regain and uncontrolled eating behavior in women with more than 24 months after BS.

Uncontrolled Eating Behavior	*B*	IC 95% (Minimum, Maximum)	*p*-Value
Model 1			
Weight regain	0.285	0.017, 1.467	0.045
Model 2			
Weight regain	0.326	0.038, 1.659	0.041
Time after surgery	−0.094	−0.228, 0.122	0.546

Notes: Linear regression; dependent variable: uncontrolled eating behavior; covariate: weight regain (kg) and time after surgery (months); *B* = regression coefficient.

**Table 5:** Correlation between QOL and weight regain in women with more than 24 months after BS.

Physical Components of QOL	*B*	IC 95% (Minimum, Maximum)	*p*-Value
Model 1			
Weight regain	−0.443	−1.156, −0.300	0.001
Model 2			
Weight regain	−0.403	−1.141, −0.184	0.008
Time after surgery	−0.092	−0.136, 0.070	0.528

Notes: Linear regression; dependent variable: physical components of QOL; co-variables: weight regain (kg) and time after surgery (months). *B* = regression coefficient.

## Data Availability

The data presented in this study are available on request from the corresponding author.
